# Host STING-dependent MDSC mobilization drives extrinsic radiation resistance

**DOI:** 10.1038/s41467-017-01566-5

**Published:** 2017-11-23

**Authors:** Hua Liang, Liufu Deng, Yuzhu Hou, Xiangjiao Meng, Xiaona Huang, Enyu Rao, Wenxin Zheng, Helena Mauceri, Matthias Mack, Meng Xu, Yang-Xin Fu, Ralph R. Weichselbaum

**Affiliations:** 1 Ludwig Center for Metastasis Research, Department of Radiation and Cellular Oncology, The University of Chicago, Chicago, IL 60637 USA; 2 Present Address: Shanghai Institute of Immunology, Key Laboratory of Cell Differentiation and Apoptosis of Chinese Ministry of Education, Department of Immunology and Microbiology, Shanghai Jiao Tong University School of Medicine, 280 South, Chongqing Road, Shanghai, 200025 China; 3 Department of Radiation Oncology, Shandong Cancer Hospital Affiliated to Shandong University, Shandong Academic of Medical Science, 440 Jiyan Road, Jinan, 250117 China; 4 Department of Internal Medicine, University of Regensburg, Universitätsstrabe 31, Regensburg, 93053 Germany; 5 Department of Pathology, University of Texas Southwestern Medical Center, Dallas, TX 75390 USA

## Abstract

Radiotherapy induces and promotes innate and adaptive immunity in which host STING plays an important role. However, radioresistance in irradiated tumors can also develop, resulting in relapse. Here we report a mechanism by which extrinsic resistance develops after local ablative radiation that relies on the immunosuppressive action of STING. The STING/type I interferon pathway enhances suppressive inflammation in tumors by recruiting myeloid cells in part via the CCR2 pathway. Germ-line knockouts of CCR2 or treatment with an anti-CCR2 antibody results in blockade of radiation-induced MDSC infiltration. Treatment with anti-CCR2 antibody alleviates immunosuppression following activation of the STING pathway, enhancing the anti-tumor effects of STING agonists and radiotherapy. We propose that radiation-induced STING activation is immunosuppressive due to (monocytic) M-MDSC infiltration, which results in tumor radioresistance. Furthermore, the immunosuppressive effects of radiotherapy and STING agonists can be abrogated in humans by a translational strategy involving anti-CCR2 antibody treatment to improve radiotherapy.

## Abstract (web summary)

Tumors often develop resistance to radiotherapy. Here the authors show that irradiation leads to a CCR2-dependent infiltration by myeloid derived suppressor cells that promote radio-resistance through inhibition of adaptive immune responses and that the use of CCR2 antibodies in mice reduces such resistance.

## Introduction

Radiation therapy is a widely employed anti-cancer treatment and is utilized in 50–60% of cancer patients^1^. The anti-tumor response elicited by irradiation (IR) depends on the innate and adaptive immunity of the host^2–5^ in which type I interferon (IFN) production and signaling play a pivotal role. Following IR, the tumor microenvironment undergoes changes including an increase in DNA damage followed by enhancement of the DNA sensing pathway via cGAS/STING, which leads to an increase in type I interferon production and signaling, and a subsequent, powerful adaptive immune response^6,7^. In cellular terms, radioresistance is defined as the doseslope or the survival cure; however, the radioresistance of tumors is multifactorial and may result from intrinsic cellular radioresistance or tumor microenvironmental factors such as hypoxia^8^. Therefore, experimental tumor radioresistance is defined as a comparatively rapid regrowth of tumor or a decrease in the number of tumors expected to be controlled at a specific dose. Radioresistant tumors are a major barrier to successful cancer treatment. For example, in locally advanced lung cancer and non-HPV head and neck cancer, patients who receive radiotherapy fail locally frequently (>50%), likely due to radioresistance, which is determinative in part of treatment success. Recently, radiation has been used in combination with immunotherapy in various clinical trials, predominantly with checkpoint inhibitors to re-invigorate T cells^9^. Data from pre-clinical models and clinical trials that are underway suggest that activation of the STING-mediated DNA sensing pathway and type I interferon production in combination with radiation and other therapies is an effective approach to cancer therapy^5,10^.

However, the roles of type I interferon in tumor immunology could be multi-faceted. Despite the importance of IFN in DC function and T cell priming for initiating anti-tumor host response, it has been noted that chronic interferon exposure can be immunosuppressive in viral infection models in that blockade of type 1 interferon signaling can reduce inflammation caused by infection^11,12^. The negative effect of type I interferon in cancer immunotherapy merits further investigation. We hypothesized that activation of STING by radiation or using STING agonists alone would be a more effective approach when combined with ameliorating the suppressive tumor microenvironment in the host.

Therapeutic radiation leads to injury-like inflammation locally that induces inflammatory responses^13^ that are anti-tumor in nature but also immunosuppressive. These immunosuppressive pathways include recruitment of myeloid-derived suppressor cells (MDSCs)^14^ and regulatory T cells (Tregs)^15^. In mice, MDSCs are identified as monocytic (M-)MDSCs (CD11b^+^Ly6C^hi^Ly6G^–^) and polymorphonuclear (PMN-) MDSCs (CD11b^+^Ly6C^lo^Ly6G^+^), respectively^16,17^. In some tumor models, M-MDSCs express higher levels of F4/80, CD115, 7/4, and CCR2.

CCR2 is a receptor for monocyte chemoattractant proteins 1, 3, and 5 (CCL2, CCL7, and CCL12) and is expressed on the surface of a subset of M-MDSCs. CCR2 ligands, CCL2, CCL7, and CCL12, are produced by various cell types, including cancer cells. CCR2^+^ cells are also important in tissue repair/remodeling due to their vessel-promoting properties^18,19^. CCR2^+^ endothelial cells play a prominent role in tumor cell metastasis^20^. In addition, CCR2^+^ M-MDSCs commonly found in various types of cancers can facilitate tumor cell extravasation and metastatic outgrowth^20–22^. A mouse monoclonal antibody to CCR2 has been developed and has shown excellent efficacy in blocking CCR2^+^ cell trafficking^23^. Selective depletion of this specific monocyte subpopulation through engagement of CCR2 by this antibody can reduce central nervous system autoimmunity^24^. Mouse anti-CCR2 has been evaluated for the treatment of inflammatory and infectious diseases, as well as rheumatoid arthritis and atherosclerosis. However, the usage of CCR2-depleting antibody has not been previously tested in cancer immunotherapy.

In this report, we demonstrate that MDSC recruitment and tumor radioresistance rely on CCR2^+^ cells in the host. Through the use of CCR2 knockout mice or an antibody against mCCR2, we observed that the anti-tumor response as a result of T cell priming was increased in mice treated with radiation, STING agonist, or both. We report for the first time that the STING pathway triggers an influx of MDSCs post-radiation; promoting the level of STING/type I IFN pathway activation also increased MDSC levels. Depletion of CCR2^+^MDSC cells enhanced the therapeutic effect of radiation and a STING agonist, as well as combined radiation plus STING agonist therapy, by decreasing suppression of T cells in the tumor microenvironment. Our results suggest that the STING/DNA sensing pathway exerts opposite, immunostimulatory effects followed by compensatory immunosuppressive effects in the tumor microenvironment. We propose that alleviating suppression as well as promoting T cell priming is equally important to the success of combined radio-immunotherapy.

## Results

### Monocytic myeloid cells accumulate in irradiated tumors

**Fig. 1:**
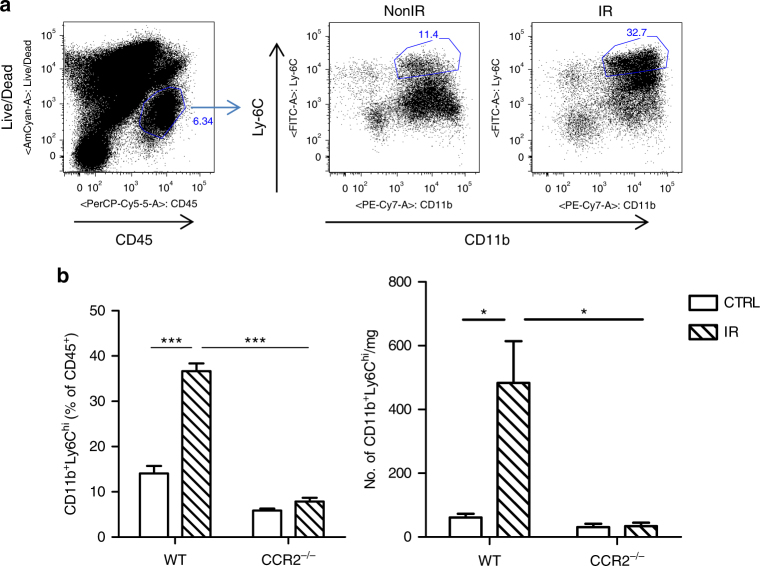
Monocytic-MDSCs are accumulated in tumors following radiation in WT but not in CCR2^−/−^ mice. Tumors were harvested 3 days post-IR and subjected to flow cytometry analysis. **a** Gating strategy and **b** flow analysis of Ly6C^hi^ populations in control (NonIR) and irradiated (IR) tumors grown in WT or CCR2^−/−^ hosts (*n* = 4). **b** Left, cell percentage; Right, absolute cell number. ***P = 0.0001; *P < 0.05, as calculated by two-sided Student’s *t*-test. The experiments were repeated 3 times. Data are presented as mean ± s.e.m

To investigate the effects of IR on the suppressive immune contexture in a tumor model syngeneic to C57BL/6 mice, we profiled the myeloid cell composition in irradiated MC38 colon tumors. 3 days after treatment with 20 Gy, the percentage of monocytic Ly6C^hi^ myeloid cells (CD11b^+^) among total hematopoietic cells (CD45^+^) was increased by as much as 3-fold compared to the percentage of Ly6C^hi^ cells in tumors of non-irradiated controls (Fig. 1a, b, *P* = 0.0001: Student’s *t*-test). In contrast, dendritic cell (CD11b^+^/CD11C^+^, CD8α^+^/CD11C^+^) levels were unchanged (Supplementary Fig. 1A, *P* > 0.05: Student’s *t*-test). We observed a decrease in macrophages (F4/80^+^) post-IR (Supplementary Fig. 1A, *P* < 0.001: Student’s *t*-test). These results suggest that local IR changes the landscape of the inflammation profile of experimental colon cancer. The increased levels of Ly6C^hi^ myeloid cells suggest the hypothesis that monocytic MDSCs may play a prominent role in rescuing irradiated tumors from the anti-tumor effects of radiation.

### Monocytic myeloid cells mediate radio-resistance

Ly6C^hi^ myeloid cells express high levels of CCR2, a chemokine receptor which enables trafficking of subsets of myeloid cells exiting the bone marrow^19^. We employed CCR2 germ-line knockout mice to investigate the involvement of CCR2^+^Ly6C^hi^ cells in the tumor radiation response. To investigate the monocytic MDSC status in CCR2^−/−^ mice, we profiled the myeloid cell population in MC38 tumors 3 days post IR. We observed that in a MC38 tumor model, the majority of CD11b^+^Ly6C^hi^ cells are CCR2^+^, in that the baseline level of the CD11b^+^Ly6C^hi^ population was lower in tumors grown in CCR2^−/−^ mice compared with tumors grown in WT mice (Fig. 1b). Following IR, the CD11b^+^Ly6C^hi^ population in tumors in CCR2^−/−^ hosts remained low (CCR2^−/−^ non-IR 5.9 ± 0.5 vs. CCR2^−/−^ IR 7.8 ± 0.8, Fig. 1b), in contrast to tumors in WT mice, where a significant increase was observed (non IR 14 ± 1.67 vs. IR 36.6 ± 1.7, in the percentage of CD45^+^ cells, *P* = 0.0001: Student’s *t*-test). Other populations of hematopoietic cells are also lower in tumors in CCR2^−/−^ hosts compared to WT hosts, such as CD11b^+^CD11c^+^ dendritic cells and CD11b^+^F4/80^+^ macrophage cells, which all may be derived from the differentiation of infiltrating monocytes or rely on CCR2 expression for circulation and trafficking (Supplementary Fig. 1B). CD11b^+^Ly6G^+^ levels in tumors of CCR2^−/−^hosts were higher than those in WT hosts, which implies a possible compensatory mechanism in the absence of CCR2 signaling.

**Fig. 2:**
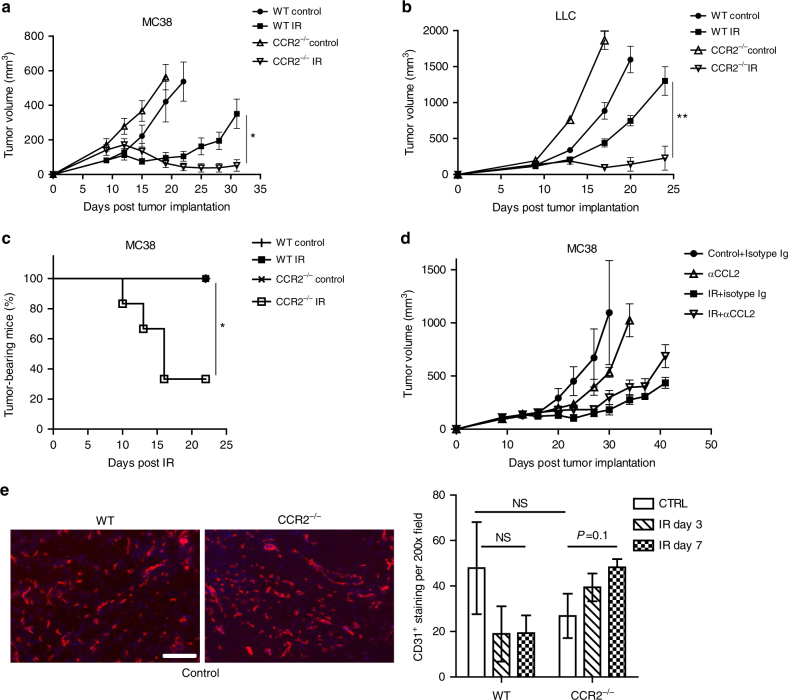
CCR2 expression in the host is crucial for monocytic-MDSC mediated tumor resistance to radiation. CCR2 knockout sensitized tumor to radiation treatment. Tumors grown in CCR2^−/−^ mice were more radiosensitive than tumors in WT mice, in both the **a** MC38, and **b** LLC tumor models. **c** CCR2 deficiency in hosts led to eradication of 60% of tumors by IR. **d** Neutralization of CCL2 could not achieve better tumor control by IR. **e** CD31 staining (left) and quantification (right) of tumors grown in WT or CCR2^−/−^ mice. Tumors were harvested and frozen sections were fixed and stained. CD31 positive cells in three DAPI positive fields from three mice were counted. Scale bar = 100 µm; **P < 0.01; *P < 0.05. Data are presented as mean ± s.e.m

We observed significantly enhanced tumor regression of MC38 tumors grown in CCR2^−/−^ hosts treated by local IR as compared with tumors grown in wild-type (WT) mice (Fig. 2a, Day 31, CCR2^−/−^ IR 52 ± 33 vs. WT IR 351 ± 82, *P* < 0.05: Student’s *t*-test). Irradiation of the tumors grown in a CCR2^−/−^ host also led to a complete response (rejection rate) of over 60% (Fig. 2c), which was not found in irradiated tumors in WT hosts (*P* < 0.05: Student’s *t*-test). We harvested frozen sections of the tumors grown in WT and CCR2^−/−^ mice and stained the vasculature. We did not observe a reduction in vessel density in either untreated control or irradiated tumor in CCR2^−/−^ hosts, compared with WT mice (Fig. 2e). This ruled out the possibility that a stronger anti-tumor effect of IR in CCR2^−/−^ mice was due to poor tumor vasculature. The potential importance of CCR2-expressing cells in radiation therapy is also observed in the Lewis Lung Carcinoma (LLC) tumor model, where LLC tumors grown in CCR2^−/−^ hosts showed significant regression post-IR compared to LLC grown in WT mice (Fig. 2b, CCR2^−/−^ IR 228 ± 166 vs. WT IR 1300 ± 199, day 24, *P* < 0.01: Student’s *t*-test). These results suggest that CCR2^+^ MDSCs contribute to tumor relapse after radiation therapy, and indicate that CCR2 is a therapeutic target for improvement of radiation efficacy. Taken together, we established a relationship between CCR2-expressing myeloid cells of hosts and tumor radioresistance.

**Fig. 3:**
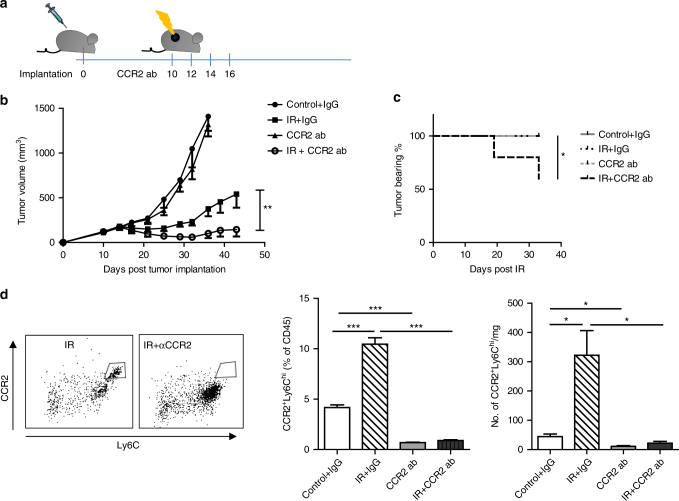
Depletion of CCR2 cells enhances tumor radiosensitivity. **a** Scheme of treatment. **b** Treated tumor was measured every 3-4 days for 33 days starting from the day of radiation (*n* = 10). **c** Percentage of mice still bearing tumor at the end of the treatments (*n* = 10), *P* < 0.05. **d** Flow cytometry profile of CCR2^+^Ly6C^hi^ in tumors 3 days post the start of treatments. Left, representative flow graph, gated on CD45^+^CD11b^+^cells; Middle, percentage of CD45^+^; Right, absolute number of cells (*n* = 4). NS, non-significant.*P < 0.05; **P < 0.01; ***P < 0.001. Each experiment was repeated 3 times. Data are presented as mean ± s.e.m

To further examine whether the involvement of CCR2-expressing myeloid cells in radioresistance can be translated into a clinically relevant strategy, we attempted to neutralize CCL2 in tumor studies because it is one of the chemokines proposed to attract CCR2^+^ cells to tissues and tumors. Fig. 2d shows that administration anti-CCL2 antibody via intratumoral injection did not enhance the anti-tumor effect of IR. Similar results were found using i.p. administration of anti-CCL2 ab and 20 Gy IR in CCL2 knockout hosts and WT mice (Supplementary Fig. 2D). Since radiation induces expression in all chemokines (CCL2, CCL7 and CCL12) that are proposed to be ligands of CCR2 (Supplementary Fig. 2A), we sought to block the receptor CCR2, which would be an effective translational strategy. Studies using a CCR2 antibody (MC21)^23^ were performed on tumor-bearing WT mice (treatment schedule, Fig. 3a). These experiments also clarified whether the enhanced efficacy of radiation on tumor regression in CCR2^−/−^ mice was due to a congenic intrinsic development defect in trafficking and maturation of myeloid-derived cells in the knockout mouse. As shown in Fig. 3b, treatment with anti-CCR2 antibodies alone had little effect on tumor regression, similar to untreated controls. However, when combined with radiation, anti-CCR2 significantly enhanced the efficacy of tumor regression compared to IR alone (144 ± 71 vs. 540 ± 151 on day 43, *P* < 0.01: Student’s *t*-test). 40% of tumors were completely rejected in the IR + anti-CCR2 treatment group (Fig. 3c). Our results demonstrate that CCR2 blockade in established tumors reverses radiation resistance and achieves greater anti-tumor efficacy.

We profiled the tumor myeloid cell population on day 3 post-IR for all treatment groups: IgG isotype control, 20 Gy + IgG control, anti-CCR2 antibody, and 20 Gy + anti-CCR2 antibody (Fig. 3d and Supplementary Fig. 1C). Of note, treatment with the anti-CCR2 monoclonal antibody (mAb) clone MC21 alone decreased the accumulation of the CCR2^+^Ly6C^hi^ population but not Ly6C^−^ or Ly6^int^cells (Fig. 3d middle and right). Compared to germ-line CCR2 knockouts (Fig. 1b), blockade of CCR2 reduced the CCR2^+^Ly6^hi^ population to a similar degree (Fig. 3d, *P* < 0.05: Student’s *t*-test). We also observed a significant increase in the CD11b^+^Ly6G^+^ neutrophil percentage in the IR + anti-CCR2 treatment group, compared with anti-CCR2 treatment or IR alone (Supplementary Fig. 1C, *P* < 0.001: Student’s *t*-test). In contrast, neither the CD11b^+^CD11c^+^ nor the CD11b^+^F4/80^+^ populations in the anti-CCR2 or IR + anti-CCR2 treatment groups changed significantly, compared to IgG isotype controls or irradiated mice, respectively (Supplementary Fig. 1C, *P* > 0.5: Student’s *t*-test). These results suggest that by specifically targeting CCR2^+^Ly6C^hi^ cells, when combined with radiation, anti-CCR2 antibody can be a potentially powerful cancer therapy without affecting differentiation and maturation of macrophages and DCs.

### M-MDSCs regulate tumor-specific CD8^+^ T cell response post-IR

**Fig. 4:**
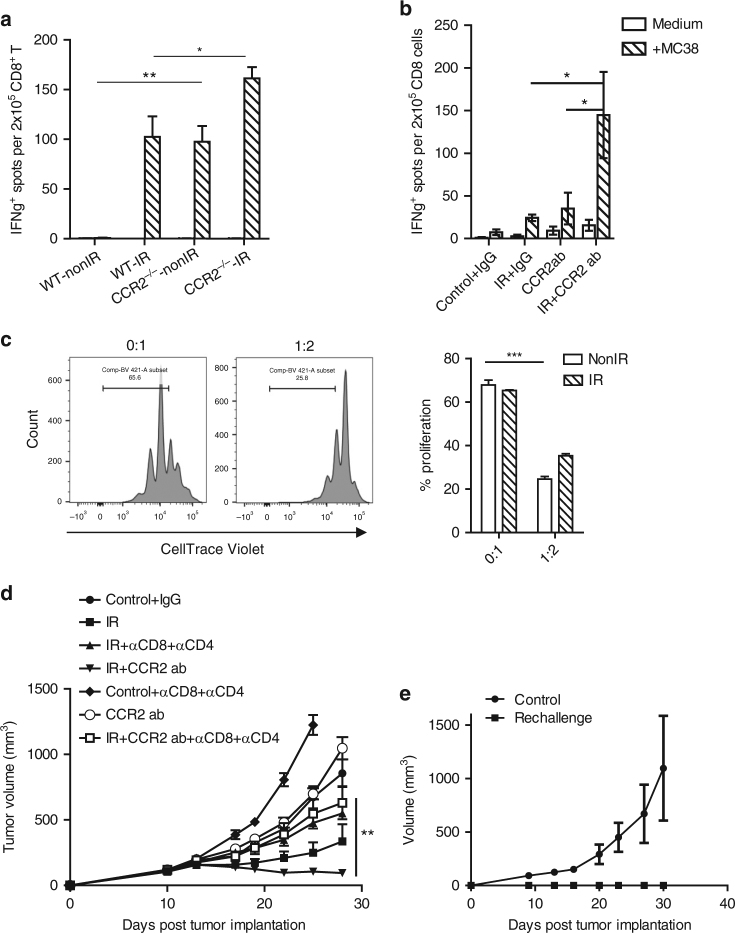
Depletion of CCR2^+^Ly6C^hi^ cells enhances adaptive T cell response, which is pivotal for the efficacy of IR + CCR2 ab treatment. **a** CD8^+^ T cells were purified from draining lymph nodes from WT and CCR2^−/−^ tumor bearing mice or WT mice that received indicated treatments, and **b** subjected to ELISPOT assay. **c** CCR2^+^Ly6c^hi^ cells were sorted from excised tumors and subjected to suppression assay. Ratio indicates CCR2^+^Ly6c^hi^: CD8. **d** Depleting T cells abolished the therapeutic effect of the IR + anti-CCR2 treatment. **e** Mice that completely rejected MC38 tumors were re-challenged two months later by five million of MC38 cells in the flanks (*n* = 5); *P < 0.05; **P < 0.01; ***P < 0.001; Each experiment was repeated three times. Data are presented as mean ± s.e.m

We and others have reported previously that host adaptive immunity (CD8^+^ T cells) is crucial for radiation-induced anti-tumor effects^2–4,25^. We sought to examine the extent to which monocytic MDSCs participate in the adaptive immune response of irradiated tumors. We performed Elispot assays for IFNγ secreting capacity of CD8^+^ T cells derived from the draining lymph nodes of tumor-bearing WT or CCR2^−/−^ mice 7 days post-IR, using MC38 cancer cells as sources of tumor antigen. There was a significantly higher level of MC38-antigen dependent IFNγ production (i.e., T cell priming) in CCR2^−/−^ mice than that of WT in the untreated control group (baseline levels, Fig. 4a, *P* < 0.01: Student’s *t*-test). T cell priming in the irradiated tumors of CCR2^−/−^ hosts was further enhanced compared to that of irradiated WT mice (Fig. 4a, *P* < 0.05: Student’s *t*-test). To rule out the possibility of intrinsic impairment of CCR2^−/−^ mice in priming, we performed ELISPOT in anti-CCR2 + IR studies. The T cell priming capacity of anti-CCR2 treated mice was slightly higher than IgG isotype control-treated mice. Most importantly, when combining anti-CCR2 with IR treatment, tumor antigen-specific T cell priming was enhanced to a greater extent compared with either IR alone (Fig. 4b, *P* < 0.05: Student’s *t*-test) or CCR2 antibodies alone (Fig. 4b, *P* < 0.05: Student’s *t*-test). The enhanced T cell function in CCR2^−/−^ mice after tumor irradiation as well as after anti-CCR2 + IR treatment indicated that CCR2^+^Ly6c^hi^ monocytic MDSCs play an important role in T cell suppression in irradiated tumors. To examine the function of the depleted cells in anti-CCR2 and radiation treatment, we assayed sorted CCR2^+^Ly6c^hi^ cells from untreated or irradiated tumors for their T cell suppression function, with sorted CCR2^−^Ly6C^int^ as control (Fig. 4c). Indeed, the CCR2^+^Ly6c^hi^ cells that could be efficiently depleted using anti-CCR2 antibody exhibited the highest CD8 T cell suppression capacity.

We performed tumor growth studies using IR + anti-CCR2 treatment while depleting CD8^+^ and CD4^+^ T cells (Fig. 4d). The efficacy of the treatment was abolished in the T cell depletion group compared with IR + anti-CCR2 treatment (*P* = 0.01: Student’s *t*-test). In addition, mice with a complete response to IR + anti-CCR2 treatment were resistant to tumor re-challenge (Fig. 4e), further confirming the establishment of tumor-specific adaptive memory response. Our results demonstrated that CCR2^+^Ly6C^hi^ monocytic myeloid cells regulate tumor response to radiation by suppressing T cell function instead of impacting vascularization in tumors (Fig. 2e), thereby eliminating the MDSC population enhanced T cell function as well as the anti-tumor response to radiation.

### M-MDSC mobilization is dependent on STING after IR

**Fig. 5:**
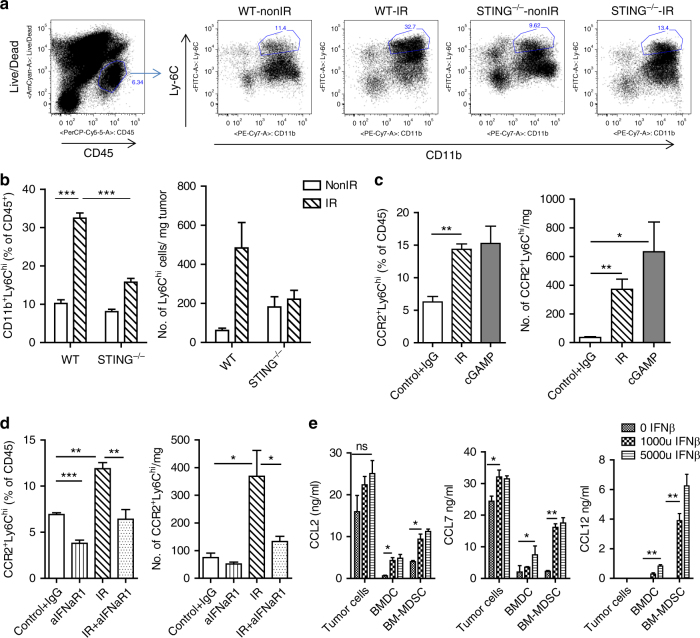
Monocytic MDSCs mobilization is dependent on STING and type I IFN signaling after irradiation. **a** Representative flow gating chart. **b** Quantification of Ly6c^hi^ population in tumors in WT and STING^−/−^ mice. **c** Activation of STING pathway by IR or by cGAMP administration can increase the recruitment of CCR2^+^Ly6c^hi^ cells. **d** Quantification of the CCR2^+^Ly6c^hi^ population in tumors grown in WT control, IR and IR + anti-IFNaR1 treated mice. **e** Type I IFN induces expression of CCL2 and CCL7 in tumor cells and CCL12 in host cells (*n* = 4); *P < 0.05; **P < 0.01; ***P < 0.001. Each experiment was repeated four times. Data are presented as mean ± s.e.m

As our results indicate, local irradiation of tumor can induce an influx of monocytic MDSCs, which in turn hampers the efficacy of IR in tumor control. We have previously reported that activation of the DNA sensing pathway in innate cells promotes IR efficacy in tumor regression; however, tumors relapse over time^5^. Here we examined whether monocytic MDSCs might compromise antitumor immunity mediated by the activation of STING, an essential component in host cell DNA sensing and the type I IFN production pathway. We implanted MC38 cancer cells on the flank of WT or STING knockout mice. After 10 days, established tumors received 20 Gy and were subjected to flow analysis 3 days later. The baseline levels of monocytic MDSCs in STING^−/−^ mice were similar to those of tumors grown in WT mice (Fig. 5a, b). The recruitment of radiation-induced monocytic MDSCs in STING^−/−^ mice was substantially lower than the level in WT mice post-IR (Fig. 5a, b, *P* < 0.001: Student’s *t*-test). We hypothesized that when radiation induces expression and activation of the STING pathway, which signals a strong DNA sensing/innate immunity reaction^5^, the signal(s) for recruitment of MDSCs is also amplified. Conversely, when STING agonist cGAMP was administered intratumorally, an accumulation of Ly6C^hi^, especially CCR2^+^Ly6C^hi^ cells, was observed at similar levels as those in irradiated tumors (Fig. 5C). STING activation triggers strong T cell priming and adaptive immunity^5^ alone and in the context of radiation. To determine whether the resulting adaptive immunity is responsible for MDSC recruitment, we conducted T cell depletion and IFNɣ neutralization studies in which tumors received local IR or cGAMP treatment. The number of CCR2^+^Ly6C^hi^ cells was not significantly reduced by the attenuation of adaptive immunity compared to their controls (Supplementary Fig. 3A and B, *P* *> *0.05: Student’s *t*-test). We observed a reduction of CCR2^+^Ly6C^hi^ as a percentage of CD45^+^ cells, but not a reduction in cell number when IFNɣ was neutralized in IR-treated tumor (Supplementary Fig. 3B). This may due to changes involving other hematopoietic cell types in these tumors. To determine the impact of the STING-DNA sensing pathway on mobilization of monocytic MDSCs in terms of function of the downstream pathway (such as type I IFN signaling), we neutralized receptors of type I IFN using an IFNaR antibody and profiled CCR2^+^Ly6C^hi^ cells in established tumors treated with local IR or IR + anti-IFNaR1 antibody. In tumors not treated with IR, anti-IFNaR1 reduced the percentage of CCR2^+^Ly6C^hi^ cells compared to controls. While IR induced a robust CCR2^+^Ly6C^hi^ cell accumulation in MC38 tumors (Fig. 5d, IR vs. non-IR, *P* < 0.01: Student’s *t*-test), blockade of type I IFN signaling abolished the induction (Fig. 5d, IR vs. IR + anti-IFNaR1, *P* = 0.01: Student’s *t*-test). STING is an essential regulator of IFN production. We propose that STING regulates monocytic MDSC recruitment at least in part through type I IFN signaling. Additionally, IFNβ induces expression of CCL2, CCL7 in tumor cells, as well as CCL12 in host cells at the transcription level (Supplementary Fig. 4A). CCL2, CCL7, and CCL12 levels were also elevated in the co-culture media when IFNβ was present (Fig. 5e) and in tumors injected with Ad. IFNβ (Supplementary Fig. 4B). This result, together with IFNaR depletion data, suggests that type I IFN production by activated STING pathway induced CCL2, CCL7, and CCL12 expression, which in turn mobilizes monocytes into tumor.^26^

Considering the decreased monocyte mobilization in irradiated tumors grown in STING knockout mice, our results suggest that the STING pathway has an immunosuppressive arm following activation by radiation or by a STING agonist, which induces monocyte accumulation, thus an adaptation of tumors to radiation. This is likely mediated in part by chemokine inductions by interferon signaling in tumor and host cells.

### Modulation of innate sensing and M-MDSC abolishes radioresistance

**Fig. 6:**
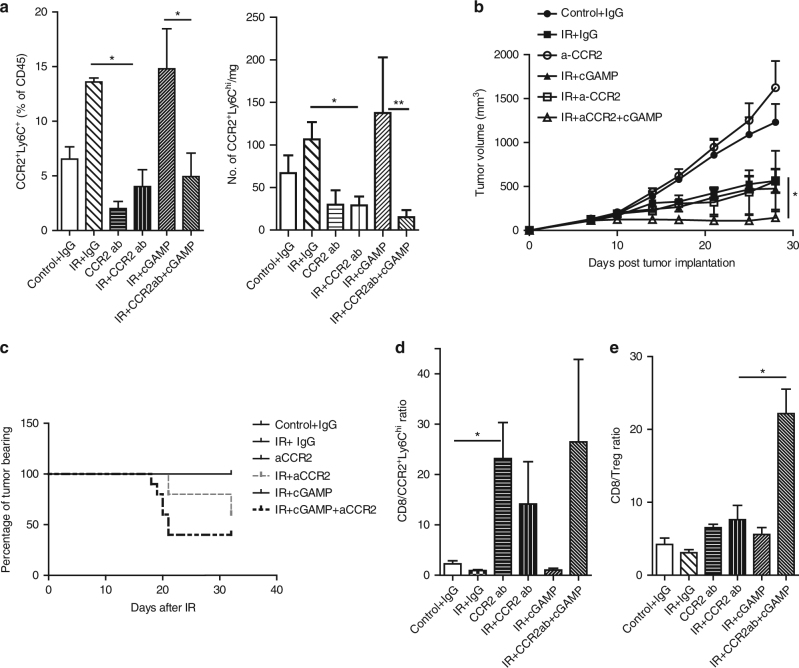
MDSC depletion enhances tumor response to radiation and STING agonist cGAMP treatment. **a** Flow cytometry analysis of immune CCR2^+^Ly6c^hi^ cells 3 days after IR (*n* = 3). **b** Tumor growth curves; *n* = 6; **c** percentage of tumor bearing mice ending on 33 days after starting of the treatments. **d**, **e** ratios of percentage of CD8 over CCR2^+^Ly6c^hi^ and Treg cells in treated tumors, respectively. *n* = 3. *P < 0.05; **P < 0.01. The experiments were repeated three times. Data are presented as mean ± s.e.m

We previously demonstrated that cGAMP, a product of cGAS and an agonist of STING, synergizes with IR to trigger a robust anti-tumor effect by enhancing the tumor-specific T cell response in the host. However, there was tumor relapse in some animals, presumably due to immunosuppression induced by interferon stimulation via influx of CCR2^+^Ly6C^hi^ monocytic MDSC. We sought to investigate whether administration of anti-CCR2 further enhances the radiation response of IR + cGAMP by abrogating MDSCs. First, we confirmed that in tumors treated by IR + cGAMP, there was an accumulation of CCR2^+^Ly6c^hi^ cells compared to non-IR control, similar to that of treatment with IR alone (Fig. 6a, *P* *< *0.05: Student’s *t*-test). Administration of anti-CCR2 antibody combined with IR + cGAMP consistently depleted CCR2^+^Ly6c^hi^ population in tumors, compared to IR + cGAMP (Fig. 6a, *P* < 0.05: Student’s *t*-test). In tumor regression studies, triple treatment of anti-CCR2 + IR + cGAMP showed superior anti-tumor response compared with treatment with IR alone or IR + cGAMP (Fig. 6b, *P* *< *0.05 for both: Student’s *t*-test). Furthermore, triple treatment rejected 60% tumors at the end of treatment (Fig. 6c, Day 22 post-IR), whereas approximately 40% of tumors were rejected by IR + anti-CCR2 treatment. These results indicate that depleting monocytic MDSCs during IR + STING agonist treatment, which triggers accumulation of MDSCs, can further enhance radiation-induced anti-tumor immunity. A similar result was observed in a LLC tumor model (Supplementary Fig. 5). In addition, the ratios of CD8^+^ to MDSCs are increased in tumors that received treatments of anti-CCR2, anti-CCR2 + IR and anti-CCR2 + IR + cGAMP compared with control, IR alone, and IR + cGAMP (Fig. 6d, *P* < 0.05: Student’s *t*-test). The CD8^+^/CD4^+^FoxP3^+^(Treg) ratio in tumors treated by the triple treatment was elevated dramatically compared to the level elicited by any other treatments (Fig. 6e, *P* < 0.05: Student’s *t*-test). Taken together, our results suggest that combining monocytic-MDSC depletion and STING activation with IR leads to a long-lasting therapeutic effect in established tumors. In addition to alleviating immunosuppression mediated by MDSC, further increases in CD8^+^/Treg ratio by adding anti-CCR2 to combined IR + cGAMP treatment, alleviating suppression of a different type, may be responsible for the superior anti-tumor immunity we observed.

## Discussion

Recent advances in the effort to increase cures by radiotherapy have recognized the importance of the immune system as central to these strategies. In addition to promoting T cell priming, radiation induces recruitment of suppressive immune cells such as MDSCs and Tregs into the tumor microenvironment at different time points post treatment. Here we reported that an extrinsic mechanism of tumor radioresistance is regulated by host CCR2^+^ M-MDSCs (CCR2^+^Ly6c^hi^). Employing CCR2 knockout mice or CCR2-depleting antibody, we demonstrated that ameliorating the immunosuppressive effects of radiotherapy can reverse radioresistance. We also proposed that STING/DNA sensing and type I interferon signaling play an important role in recruiting M-MDSCs. Targeting M-MDSCs in combination with radiation and STING agonist treatment further enhanced anti-tumor efficacy by alleviating suppression and reducing Treg occurrence. MDSCs have been linked with tumor metastasis and tumor resistance to treatments including immune checkpoint blockade^27^. The increased level of MDSCs following SBRT (Stereotactic Body Radiation Therapy) might account for the relatively high rate of failure of high-dose ablative radiotherapy, which can be as high as 40% in large tumors. Therefore, thwarting MDSCs from being recruited into tumor immediately following radiation would be not only a very powerful complement to immune checkpoint blockade inhibitor therapy and T cell therapy, but also very effective when combined with radiotherapy. In this context, priming of the immune system can be considered radiosensitization by modifying the host, whereas infiltration of MDSCs can be considered a host adaptation to stress and therefore a new type of radioresistance.

In the CCR2 knockout study, we observed growth acceleration especially in untreated LLC tumors in CCR2 knockout mice (Fig. 2). Since CCR2^+^ monocytes are absent in the periphery of CCR2^−/−^ mice (because they are retained in the bone marrow), we hypothesize that non-MDSC (Ly6C^−^) CCR2^+^ monocytes may suppress tumor growth in certain tumor models under basal conditions because these cells may directly attack tumor cells or present tumor antigens to T cells. Because we did not directly observe MDSC mobilization in our study we cannot rule out the (unlikely) possibility that elevated levels of Ly6C^**+**^ myeloid cells are due to proliferation/conversion of progenitor cells. In studies of chemokine/receptor interactions, CCL2, CCL7, and CCL12 are described as ligands for CCR2^28,29^. Deletion of one of the CCR2 ligands, CCL2, in the host has been reported to diminish both cancer cell metastatic capacity and primary tumor growth^22,30^. However, our results did not demonstrate CCL2 blockade as an effective strategy for blocking the action of CCR2, likely because of the multiple ligands that bind CCR2 (Supplementary Fig. 2A and C). We therefore focused on the receptor CCR2 for a therapeutic target because of the difficulty of blocking multiple chemokines. These differences in the results of chemokine blockage are likely due to different tumor model systems and experimental conditions. A recent clinical trial^31^ showed that addition of a small molecule inhibitor of CCR2 to FOLFIRINOX in the treatment of advanced pancreatic cancer demonstrated anti-tumor effects. The response was proposed to be mediated by reversal of immune suppression within the tumor microenvironment^31^, which supports our strategy of combining inhibition of CCR2 and IR.

The type I interferon pathway is required for DC maturation, antigen presentation and T cell priming in numerous cancer studies^4,32^. DNA sensing cGAS/STING activation by radiation and/or STING agonist promotes type I IFN production and signaling in dendritic cells^5,7^, leading to increased priming of T cells and tumor control. Although host IR induction of type I IFN production is STING dependent, we cannot rule out the possibility that IR may regulate other pathways which leads to alteration of IFN levels. Nonetheless, by increasing type I IFN production in the tumors, we hypothesize STING activation or radiotherapy could be a “double edged sword”, which also increases immunosuppressive cytokine/chemokine production in the tumor microenvironment^33^, resulting in the influx of immunosuppressive cells.

In summary, we describe a new immunosuppressive effect of STING following radiation that results in an influx of CCR2^+^ M-MDSCs that rescue tumors from the anti-tumor effects of radiation. This host adaptation to stress has important translational implications for radiotherapy.

## Methods

### Mice and cells lines

All C57BL/6 wild-type mice were purchased from Envigo (Indianapolis, IN). CCR2^−/−^ and CCL2^−/−^ mice were purchased from The Jackson Laboratory (Bar Harbor, ME) and bred in the animal facility of The University of Chicago. STING knockout mice were a generous gift from Dr. Glen N. Barber of University of Miami School of Medicine. All the mice were used in accordance with the animal experimental guidelines set by the Institute of Animal Care and Use Committee of The University of Chicago. The MC38 and LLC cell lines were purchased from ATTC. All cell lines were authenticated and are free of mycoplasma and other common rodent viruses (tested by IDEXX).

### Tumor Growth and Treatments

1 × 10^6^ MC38 or LLC tumor cells were subcutaneously injected into the flank of female 6-8 week-old mice. Tumor volumes were measured along three orthogonal axes (*a*, *b*, and *c*) and calculated as tumor volume = *a* *b* *c*/2. Tumors were randomized by size with matched sizes in different treatment groups and treated by local IR, as described previously^5^, and tumor volumes were measured twice weekly. In brief, mice were anesthetized by Ketamine injection. Established flank tumors (100–200mm^3^ in size) were irradiated by X-ray generated from RS-2000 Biological Irradiator (RadSource) while the rest of mouse body was shielded by lead. For CCL2 neutralizing experiments, anti-CCL2 mAb (clone 2H5, BioXcell) was administered 200 μg/mouse i.p. or i.t. on 0, 2, 4, and 7 days after IR. For CCR2 depletion, control IgG or anti-CCR2 mAb (MC21^23^) was injected i.p. at 45ug/mouse at day 0, 2, 4, and 6 after IR. For CD4 and CD8 depletion, 200 μg/mouse of anti-CD8 (BioXCell, clone 53-6.7) and anti-CD4 (BioXCell, clone GK1.5) were injected i.p. on day 0, 4, and 8 post IR. Anti-IFNɣ antibody (BioXcell, clone XMG1.2) was administered i.p. at 500 μg/mouse on day 0 and 1 post-IR. For type I IFN blockade experiments, 200 μg anti-IFNαR1 mAb was intratumorally injected on day 0 and 2 after radiation. For cGAMP treatment experiments, 10 μg 2′ 3′-cGAMP in PBS was intratumorally administered on days 2 and 6 after radiation.

### Flow cytometry

Tumors were excised and diced and made into cell suspension using digesting media containing 1–2 mg/ml of Collagenase (CSL-1, Worthington Biochem., Lakewood, NJ), and 0.4 mg/ml DNase. CD45 (1 μg/ml; Cat. 45-0451-82), Ly6C (1 μg/ml; Cat. 17-4801-82, eBioscience, San Diego, CA), CD11b (1 μg/ml; Cat. 101216), Gr1 (2.5 μg/ml; Cat. 108417), Ly6G (1 μg/ml; Cat. 127624), F4/80 (1 μg/ml; Cat. 123116), CD11c (1 μg/ml; Cat. 117310), CD8 (1 μg/ml; Cat. 100708; BioLegend, San Diego, CA), CD4 (2.5 μg/ml; Cat. 553729; BD Biosciences, San Jose, CA), and CCR2 (2.5 μg/ml; Cat. FAB5538P; R&D Systems, Minneapolis, MN) were used for surface staining. Foxp3 staining was performed following the manufacturer’s instructions (Cat. 88-8111-40). Samples were analyzed on LSR-Fortessa (BD Biosciences, San Jose, CA) at The University of Chicago Flow Cytometry Core facility. At the time of flow, 30 μl of CountBright Absolute Counting Beads (ThermoFisher Scientific) were added into each sample for calculating absolute cell number. Results were analyzed using FlowJo software (Tree Star, Ashland, OR).

### Cell culture and qRT-PCR

Bone marrow cells were cultured in complete RPMI1640 with the presence of 20 ng/ml GM-CSF (DC) and GM-CSF + 10ng/ml IL6 (MDSC). 5 × 10^5^ MS38 cells, BMDC and BM-MDSCs were co-cultured with 0, 1000 and 5000U of mIFNβ1 (Biolegend, Cat. 581302) for 24 h. Cells were resuspended in Trizol (ThermoFisher). RNA was extracted and qRT-PCR was performed, as described elsewhere^34^.

The primers are listed as follows:

CCL2 5′-CACAACCACCTCAAGCAC-3′ 5′-AAGGGAATACCATAACATCA-3′

CCL7 5′-GCCTGAACAGAAACCAAC-3′ 5′-TATCCCTTAGGACCGTGA-3′

CCL12 5′-ACTTCTATGCCTCCTGCTC-3′ 5′-CACTGGCTGCTTGTGATT-3′

### ELISA

CCL2, CCL7 and CCL12 levels in culture supernatants and tumor homogenates were measured using Mouse CCL2 Duoset ELISA kit, CCL12 quantikine ELISA kit (R&D). CCL7 levels were measured using an ELISA kit (Origene, Rockville, MD and Innovative Research, Novi, MI). The quantification was normalized to volume of supernatant or weight of the tumor samples and plotted on Graphpad prism 5.0.

### IFN-γ ELISPOT and suppression assay

CD8^+^ T cells were sorted from tumor draining lymph nodes using a CD8 isolation kit (Stemcell, Vancouver, BC). 2 × 10^5^ of CD8^+^ T cells were co-cultured with 2 × 10^4^ IFN-ɣ treated MC38 tumor cells in 96-well PVDF plates (EMD Millipore, Billerica, MA) for 2 days. Spots were developed by following the manufacture’s protocol (BD Biosciences) and details have been described elsewhere^5^.

For the suppression assay, MC38 tumors were collected 3 days post-IR and the CD11b^+^CCR2^+^Ly6C^hi^ cell population was sorted using AriaIIIu 4-15 (BD Biosciences, San Jose, CA) at The University of Chicago Flow Cytometry Core facility. In the meantime, naïve CD8^+^ T cells derived from draining lymph nodes were labeled with CellTrace^TM^ Violet dye (ThermoFisher) and washed. The sorted MDSCs and 2 × 10^5^ labeled T cells were co-cultured in complete RPMI1640 with the presence of 1ug/ml αCD28 (Biolegend, clone 37.51) and 100 uM of β-mercapitoethanol (Sigma), in the wells of a flat-bottom 96-well plate coated with 5ug/ml αCD3 (Biolegend, clone 145-2C11). The amount of MDSCs was diluted by factor of 2 in serial dilutions, starting with 2 × 10^5^ (1:1). Cells were harvested, stained for CD8^+^ T cells (Biolegend, clone 53-6.7) and analyzed by flow cytometry.

### Immunofluorescence staining

Frozen sections of tumor tissues were fixed by 4% paraformaldehyde and stained with CD31-Biotin (1.6 μg/ml; Cat. NB100-1642B; Novus, Littleton, CO). After washing, sections were incubated with Alexa Fluor® 594 streptavidin(1:400; Life Technologies, Carlsbad, CA) and DAPI. Images were captured with a Zeiss Axiovert 200 m inverted epifluorescence microscope (Carl Zeiss Microscopy, Thornwood, NY) with a Hamamatsu Orca ER CCD camera (Hamamatsu Photonics, Skokie, IL) run by SlideBook 5.5 software (Intelligent Imaging Innovations, Denver, CO). Imaging was performed at The University of Chicago Integrated Light Microscopy Facility.

### Statistics

Sample sizes in all studies were determined by power analysis assuming 2-sided significance as 5% at 80% power level. In all cases, a sample number ≤5 was reached. Statistics were performed using Student’s *t*-test assuming unequal variances.

### Study approval

All animal studies and procedures were approved by the Institute of Animal Care and Use Committee of The University of Chicago.

### Data availability

The authors declare that all data are available within the Article and Supplementary Files, or available from the authors upon request.

## Electronic supplementary material

Supplementary Information
