# Supplementary material for: Host STING-dependent MDSC mobilization drives extrinsic radiation resistance
Source: Nat Commun. 2017 Nov 23;8:1736. doi: 10.1038/s41467-017-01566-5 (PMC5701019; doi:10.1038/s41467-017-01566-5)
Supplement: Supplementary file 1 — Supplementary Information [file 41467_2017_1566_MOESM1_ESM.pdf]

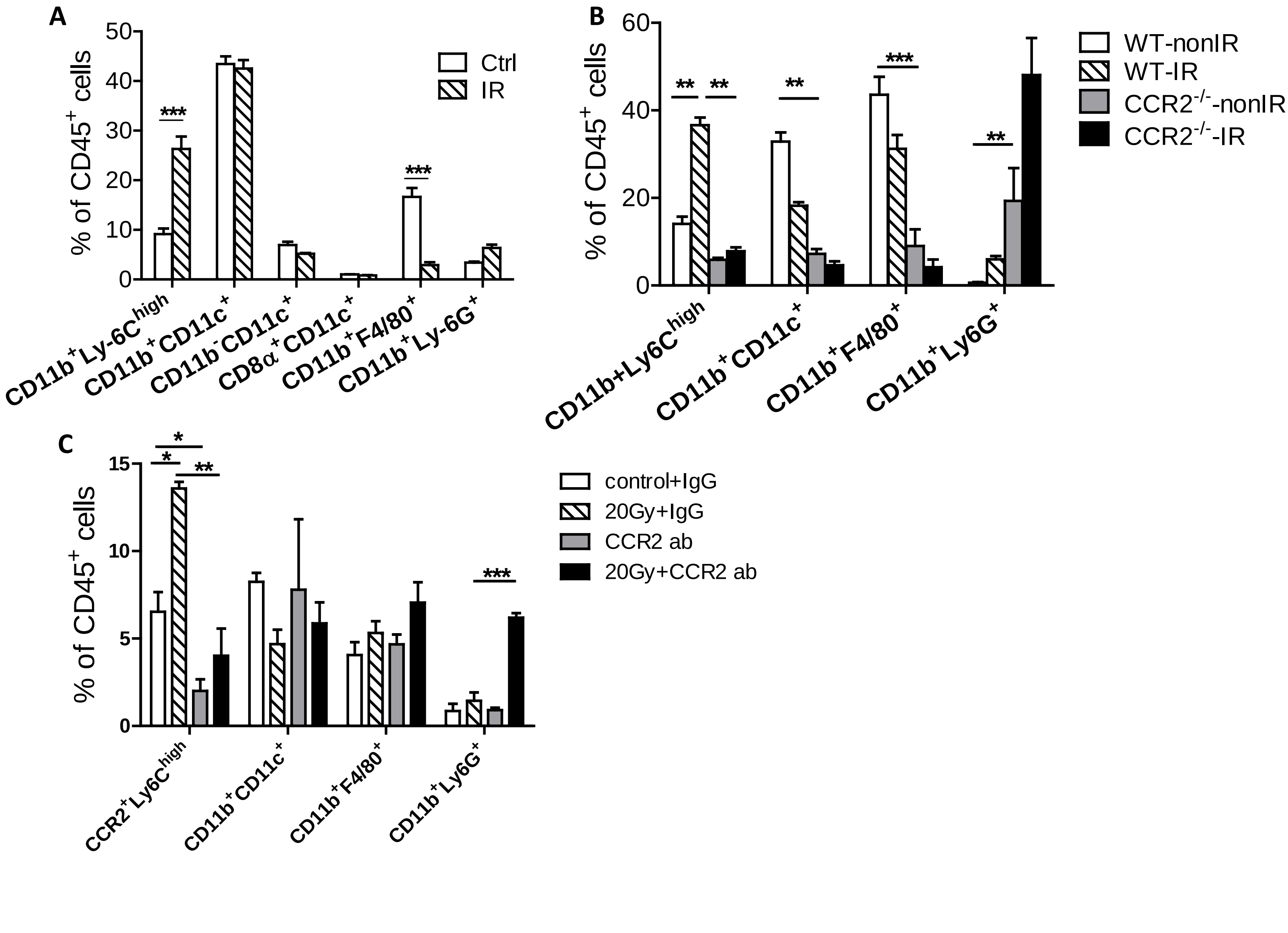

Supplementary Figure 1. Cell profiling by flow cytometry of Myeloid derived cells from MC38 tumors 3 days after irradiation. A) Cell percentages in tumors grown in WT hosts with or without IR; B) Myeloid cell percentage in tumors of WT and CCR2 knockout mice with or without irradiation. C) Myeloid cell profile in tumors of WT mice that received treatments of CCR2 depletion with or without irradiation. *, $P<0.05$; **, $P<0.01$; ***, $P<0.001$. n=4. The experiments were repeated at least 3 times. Data are presented as mean +/- s.e.m.

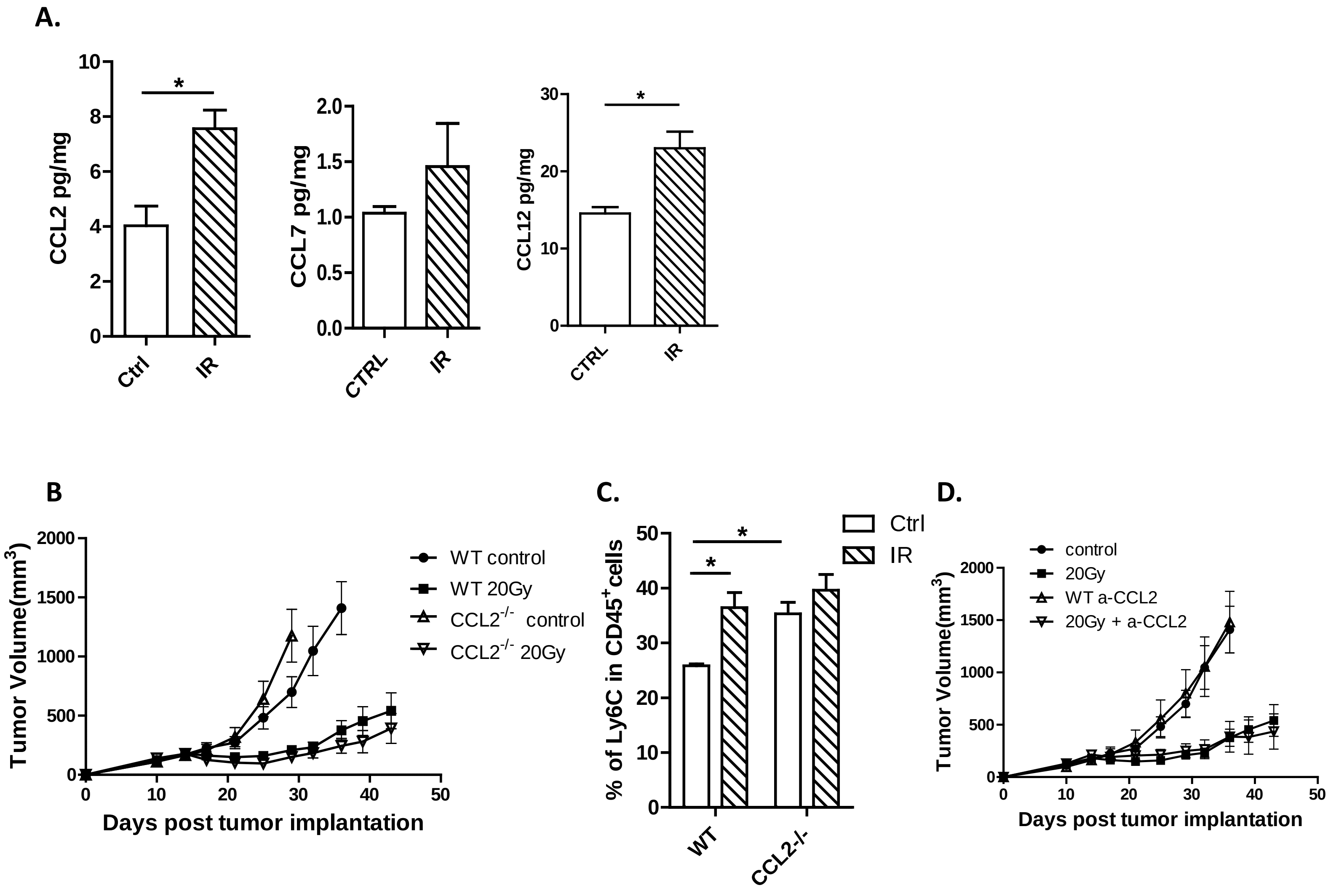

Supplementary Figure 2. Neutralizing CCL2 has no additional effect on IR-induced tumor regression. A) The chemokine levels of CCL2, CCL7 and CCL12 are elevated in tumors 3 days after local radiation. B) Tumor growth in WT or $CCL2^{-/-}$ mice after local radiation treatment showed no difference. C) The $Ly6C^{hi}$ population was not reduced in $CCL2^{-/-}$. D) On days of 0, 2, 4, and 7 relative to irradiation, 200µg/mouse anti-CCL2 antibody was administered via i.p. in mice with established MC38. Anti-CCL2 treatment showed no additional enhancement of the anti-tumor effects of IR. * P<0.05; Data are presented as mean +/- s.e.m.

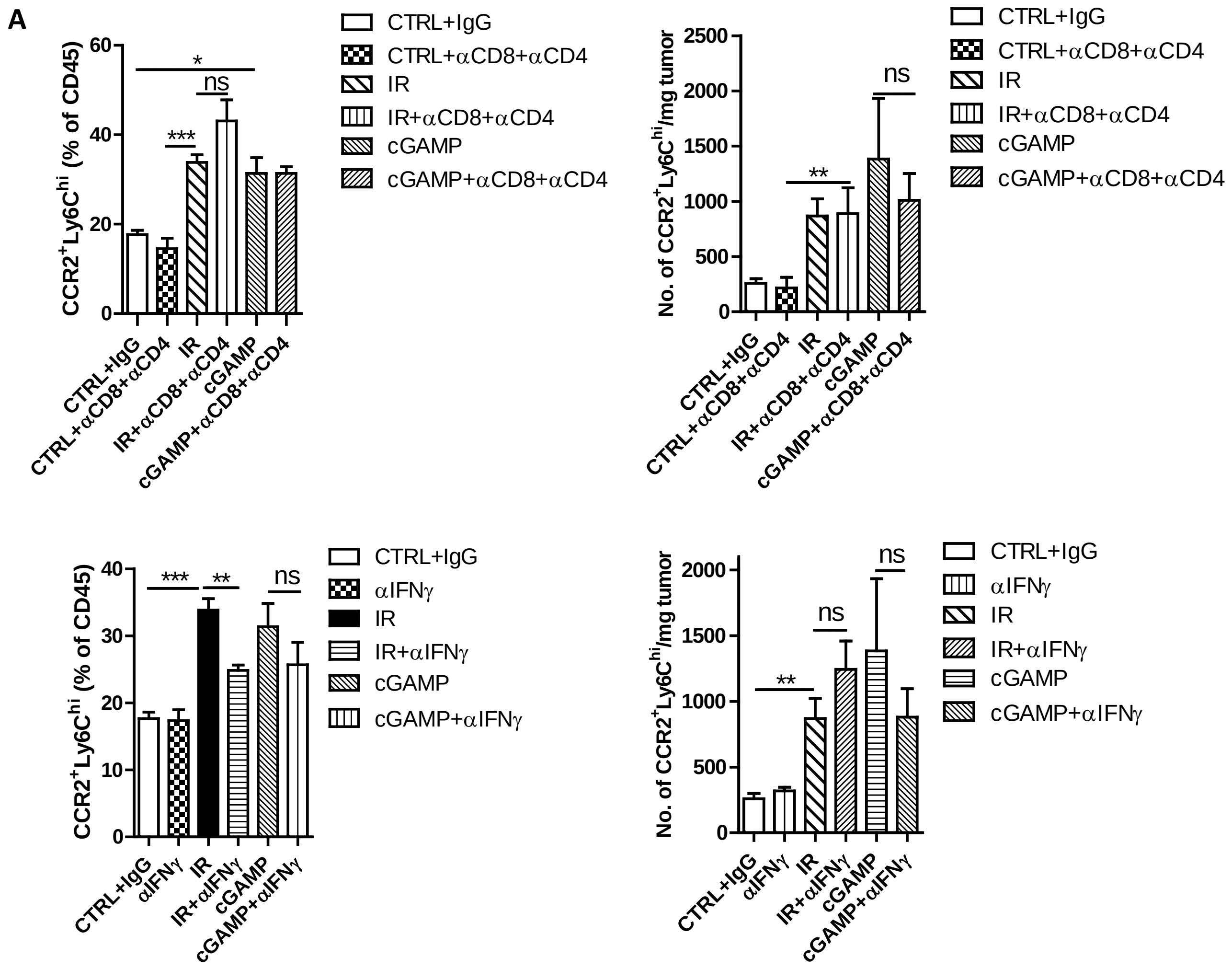

Supplementary Figure 3. **Adaptive immunity is not involved in the recruitment of CCR2$^+$MDSCs upon IR or STING activation.** 1x10^6 MC38 were injected and tumors were treated after 10 days. Three days post-treatment, tumors were harvested, stained and subjected to flow analysis. A) T cell depletion did not reduce the induction of accumulation of MDSCs by IR or cGAMP. B) Neutralizing IFNγ lowered the percentage of MDSC in the tumors induced by IR but not by cGAMP. MDSC cell number was not changed. Ns, not significant; *, P<0.05; **, P<0.01; ***, P<0.001. N=4. Experiment was repeated 2 times. Data are presented as mean +/- s.e.m.

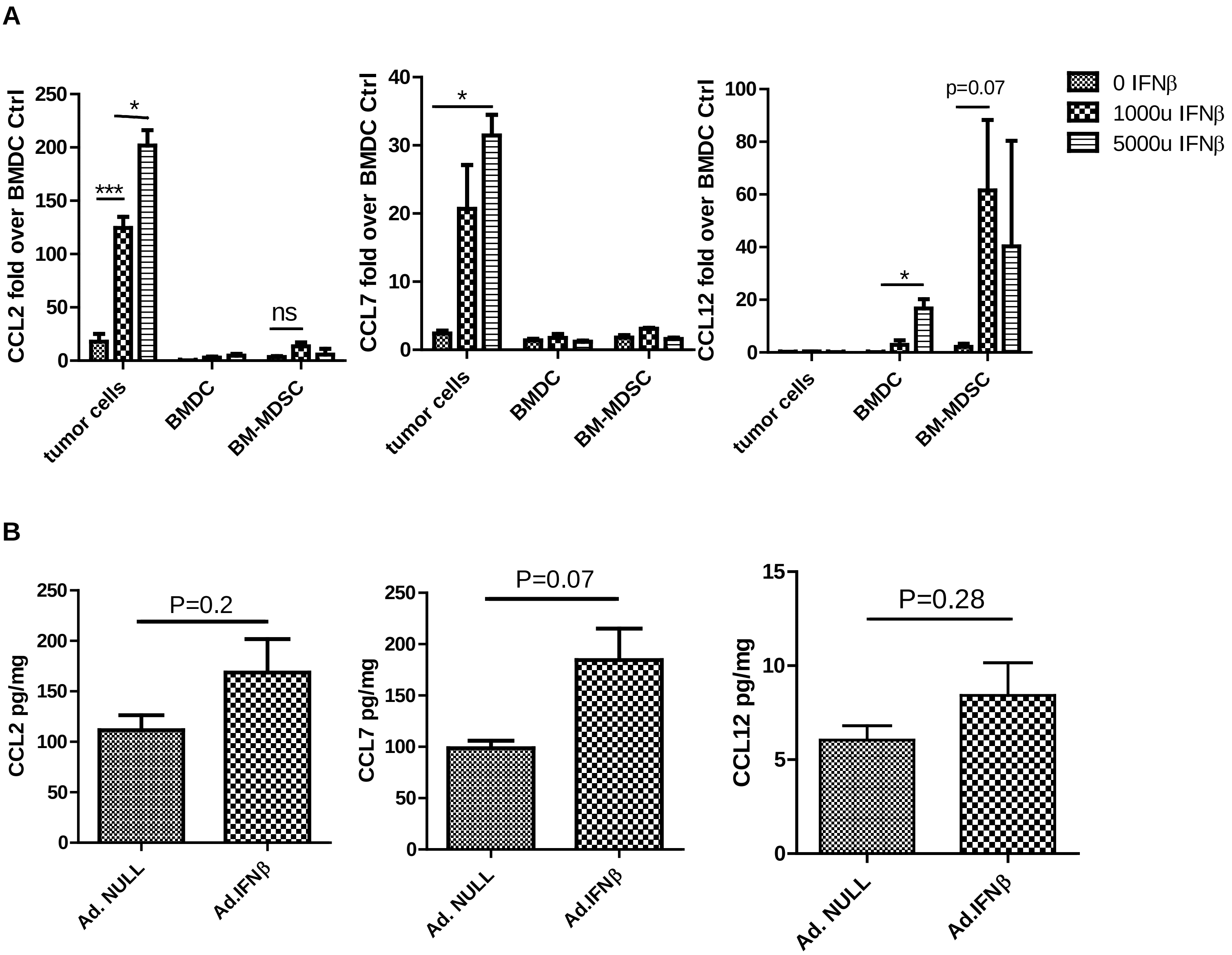

Supplementary Figure 4. **Type I IFN induces expression of CCL2, CCL7 and CCL12 in tumor cells, BMDCs and BM-MDSCs.** A) Indicated cells were cultured with mouse IFNβ for 24 hours. Real-time PCR analysis of RNA was performed. B) MC38 tumors were injected with 10^{10} VP of Ad. Null or Ad. IFNβ. Tumor homogenates were prepared 2 days later for ELISA assays. Data are presented as mean +/- s.e.m.

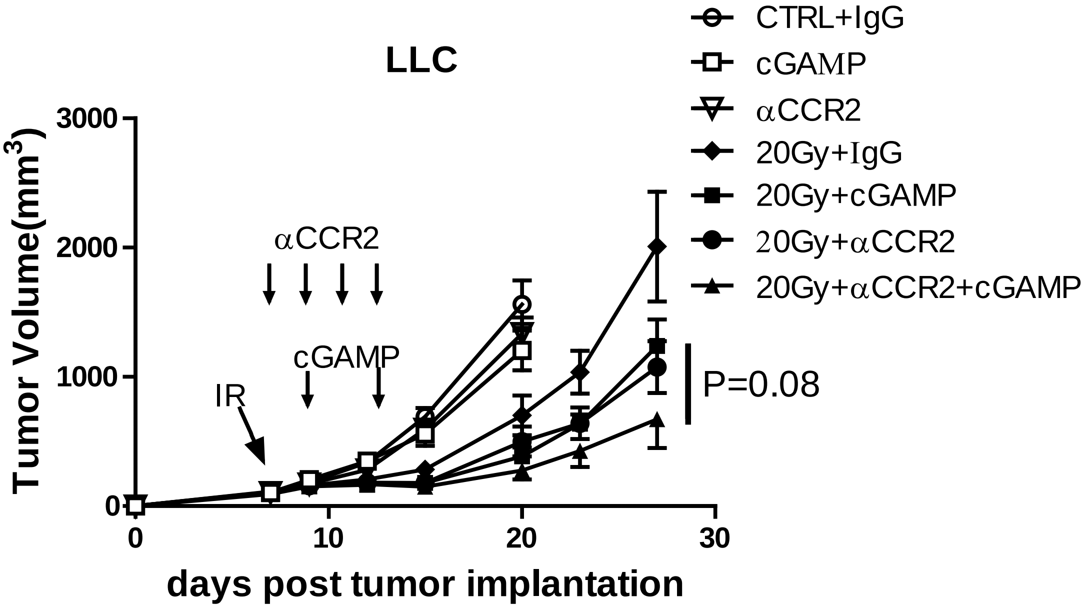

Supplementary Figure 5. Depletion of MDSCs enhances anti-tumor effect of IR and STING agonist. Treatment in the LLC tumor model. Data are presented as mean +/- s.e.m.
